# Single‐cell sequencing analysis reveals the dynamic tumour ecosystems of primary and metastatic lymph nodes in nasopharyngeal carcinoma

**DOI:** 10.1111/jcmm.70137

**Published:** 2024-10-11

**Authors:** Dahua Xu, Nihui Zhang, Yutong Shen, Dehua Zheng, Zhizhou Xu, Peihu Li, Jiale Cai, Guanghui Tian, Qingchen Wei, Hong Wang, Hongyan Jiang, Meng Cao, Bo Wang, Kongning Li

**Affiliations:** ^1^ College of Biomedical Information and Engineering Hainan General Hospital and Hainan Affiliated Hospital, Hainan Medical University Haikou China; ^2^ Hainan Engineering Research Center for Health Big Data Hainan Medical University Haikou China

**Keywords:** immune ecosystem, lymph node metastasis, NPC, single‐cell sequencing

## Abstract

Lymph node metastasis contributed to the leading cause and treatment failure in nasopharyngeal carcinoma (NPC). The microenvironment and the cellular communications of lymph node metastasized tumours determine the tumour progression and therapeutic effect, but the ecosystems about the lymph node metastasis (LNM) for NPC patients remain poorly characterized. Here, we integrated the transcriptomes of 47,618 single cells from eight samples related to NPC LNM. The dynamic immune ecosystems and immunosuppressive microenvironment including T cells, myeloid cells and B cells were observed in the lymph node metastatic samples compared with primary tumours. Additionally, the heterogeneity of epithelial cells was also revealed, and several clusters with expression programs that were associated with the progression‐free survival of NPC patients were identified. Additionally, our data revealed the complex intercellular communications from primary to lymph node metastasis. The rewiring of CCL signalling which plays an important role in tumour metastasis was further identified. Altogether, we systematically characterized the ecosystem of NPC primary and lymph node metastasized tumours, which may shed light on the development of a therapeutic strategy to improve clinical outcomes of NPC patients with lymph node metastasis.

## 1 INTRODUCTION

Nasopharyngeal carcinoma (NPC) is a cancer that originates from the nasopharyngeal epithelium and is prevalent in southeastern Asia. As a type of squamous cell carcinomas of the head and neck primaries, NPC exhibited the highest frequency for regional lymph node metastasis (LNM) among all head and neck carcinomas (HNSC),^1^ which contribute to the tumour relapse and failure of treatment. Clinical trials have demonstrated that locoregional radiotherapy could improve the overall survival of metastatic NPC patients and decrease the frequency of distant metastatic recurrences.^2^, ^3^ However, there are still a few patients with LNM who did not benefit from this regimen. Studies have found that the composition of lymph nodes undergoing remodelling impacts the cancer metastasis and treatment,^4^, ^5^ which indicates the complex tumour immune microenvironment (TIME) of lymph node metastasis with unexplored mechanisms. Therefore, it is urgent to characterize the molecular features and microenvironments of NPC primary tumours (PTs) and lymph nodes with metastasis.

The spatiotemporal communications and the cellular compositions including malignant, immune and stromal cells constitute the complex tumour ecosystems.^6^ The emerging single‐cell technology sheds light on the underlying mechanism of the tumour ecosystem, especially for tumours with lymph node metastasis. For instance, Liu et al. generate single‐cell maps of PTs and LNM tumours for breast cancer patients and demonstrate the immunosuppressive microenvironment of LNM compared to PTs.^7^ The lymph node metastatic and primary ecosystems for oesophageal squamous carcinoma are also comprehensively characterized, which highlights the crosstalk of APOC1 + APOE+ macrophages with other cells in LNM.^8^ In addition, single‐cell sequencing for gastric cancer revealed the disordering of the neutrophils polarization and activation of SPP1 contributed to the LNM.^9^ These studies have revealed the valuable capacity of single‐cell sequencing in revealing the tumour ecosystem and accelerating the discovery of biomarkers for tumour patients with lymph node metastasis. However, a global view of the tumour ecosystem in PT and LNM for NPC patients is still lacking.

In this study, we integrated the transcriptome of 47,618 single cells from normal nasopharynx tissue, lymph nodes, NPC primary tumours and NPC lymph nodes with metastasis to generate an atlas of the NPC metastatic ecosystem. We observed an immunosuppressive microenvironment of metastatic lymph nodes compared to primary tumours, which is conducive the NPC metastasis. We also investigated the prognostic value of identified cell clusters in the bulk NPC profiles. Moreover, the crosstalk of CCL signalling was found to be disrupted between PT and LNM, which may contribute to tumour metastasis. Our comprehensive study that focuses on the NPC metastatic ecosystem provided an improved understanding of the mechanism of tumour metastasis, which will aid the development of personalized therapeutic strategies for NPC patients with metastasis.

## 2 MATERIALS AND METHODS

### 2.1 Data collection

The single‐cell RNA‐seq datasets for matched primary and regional lymph node metastasis of NPC were obtained from the Genome Sequence Archive (GSA) under the accession number HRA000036.^10^ This data included two primary and four RLN metastatic samples. In addition, the single‐cell datasets for one normal nasopharynx tissue and one normal lymph node without neoadjuvant chemotherapy were obtained from the Gene Expression Omnibus (GEO) with accession number GSE150430^11^ and GSE199619.^12^ The sample information and cell numbers for single‐cell sequencing datasets were provided in Table S1. One hundred and thirteen fresh, treatment‐naïve nasopharyngeal carcinoma tumour samples with progression‐free survival (PFS) information were downloaded from GSE102349.^13^

### 2.2 Processing of single‐cell sequencing data

The raw fastq files for single‐cell sequencing data were first input to Cellranger (Version 1.1.0) with default parameters to quantify the gene expression matrix for each cell (reference: GRCh38). Then, the matrix was transformed into a Suerat object using the Seurat (Version 4.3.0.1) R package. Quality control and normalization were applied according to the Suerat pipeline. Specifically, cells with detected genes of more than 9000 as well as 10% mitochondrial gene counts were filtered. The R ‘harmony’ package was used to integrate single‐cell sequencing from different datasets to remove the batch effect.^14^ Finally, the cells were catalogued as normal nasopharynx tissue (NPC_N), primary nasopharyngeal carcinoma (NPC_PT), normal lymph node (NPC_LN) and regional lymph node metastasis of nasopharyngeal carcinoma (NPC_LNM).

### 2.3 Cell type annotation

To identify distinct cell clusters, the top 2000 variable genes were first calculated by principal component analysis. Then, the ‘FindClusters’ was performed to identify the cell clusters. The cell cluster‐specific expressed genes were identified via the Wilcoxon rank sum test via the ‘FindAllMarkers’ function. Genes with *p*‐value <0.05 and logFC >0.25 were considered differentially expressed genes (DEGs). The marker genes were obtained from the literature (Table S2), and we annotated cells based on the expression level of these marker genes in specific cell types. The clusters were annotated as five major cell types, including B cells (CD79A, MS4A1), T cells (CD2, CD3D), epithelial cells (KRT5, KRT18), myeloid cells (CD68, MS4A6A) and plasma cells (TNFRSF17, SDC1). The abovementioned clustering steps were repeated in each major cell type to identify and annotate subclusters based on the expression of marker genes (Tables S2). Moreover, the cell type scores were calculated via the top expressed 50 genes by single sample gene set enrichment analysis (ssGSEA).^15^

### 2.4 Estimation of copy number variants (CNV) and intratumoral heterogeneity for epithelial cells

The inferCNV (Version 1.10.1) was used to explore the heterogeneity of CNV in epithelial cells of NPC primary tumour and lymph node metastasis. The epithelial cells expression matrix was used as control reference, and the interCNV analysis with default parameters was performed. Then, the epithelial cells were categorized into tumour cells and normal cells according to their CNV scores.

To understand the heterogeneity of expression patterns in epithelial cells, consensus non‐negative matrix factorization (NMF) was used to deconvolute the expression matrix from the epithelial cells. We considered the trade‐off of cophenetic distance for K from 2 to 10 to obtain the optimal number for further analysis.

### 2.5 Developmental trajectory analysis

The Monocle2 was used to infer the lineage differentiation of each major cell type.^16^ The expression data from Seurat was first converted to the CellDataSet object as the input of Monocle2. Then, the differentially expressed genes between cell clusters were used to perform DDRTree‐based dimension reduction and cell ordering. The pseudotime and developmental trajectory were obtained with the default parameters of Monocle2.

### 2.6 Functional module scores and enrichment analysis

The gene sets of functional modules in each cell type were collected from previous studies,^8^, ^11^, ^17^, ^18^ including proliferation, apoptosis, cytotoxicity, IL2R, antigen processing and presentation, immune suppressive, proliferation, costimulation, exhausted and inhibitory for T cells; anti‐inflammatory, pro‐inflammatory, antigen processing and presentation (APC) and chemotaxis for macrophage cells; APC, apoptosis, costimulation, dendritic cell differentiating and immune suppressive for DC cells; immune proliferation and antigen secretion for B cells. The detailed information of these modules is provided in Table S3. The functional module scores were further calculated in each cell via the AddModuleScore function in Seurat. The correlation between module scores and cell pseudotime was calculated by Spearman's rank correlation. The functional enrichment analysis was performed based on the cell‐type top 50 expressed genes or differently expressed genes via clusterProfiler.^19^

### 2.7 Survival analysis

After excluding the samples with missing clinical information, 88 NPC patients with PFS were retained for survival analysis. Based on Jia et al.'s research method, we used the signature score of cell subpopulations to estimate their prognostic values in bulk samples.^8^ The marker gene signature (top 50 expressed) of different cell subclusters was calculated in the bulk NPC samples via ssGSEA. The median value was set as a cutoff to classify the NPC patients into high‐ and low‐expression groups. Then, Kaplan–Meier curves and log‐rank tests were performed to estimate the prognostic efficacy of different cell subtypes.

### 2.8 Cell–cell communication analysis

CellChat (Version 1.6.0) was used to estimate the potential interactions across different cell types for NPC primary and regional lymph node metastasis samples, which based on the known ligand‐receptor (LR) pairs. In addition, the dynamic changes between the primary tumour and lymph node with metastasis were compared.^20^ Differentially LR pairs between primary and metastasis were identified based on the communication probability and the one‐sided permutation test.

## 3 RESULTS

### 3.1 A single cell map of NPC primary and lymph node metastatic

**FIGURE 1:**
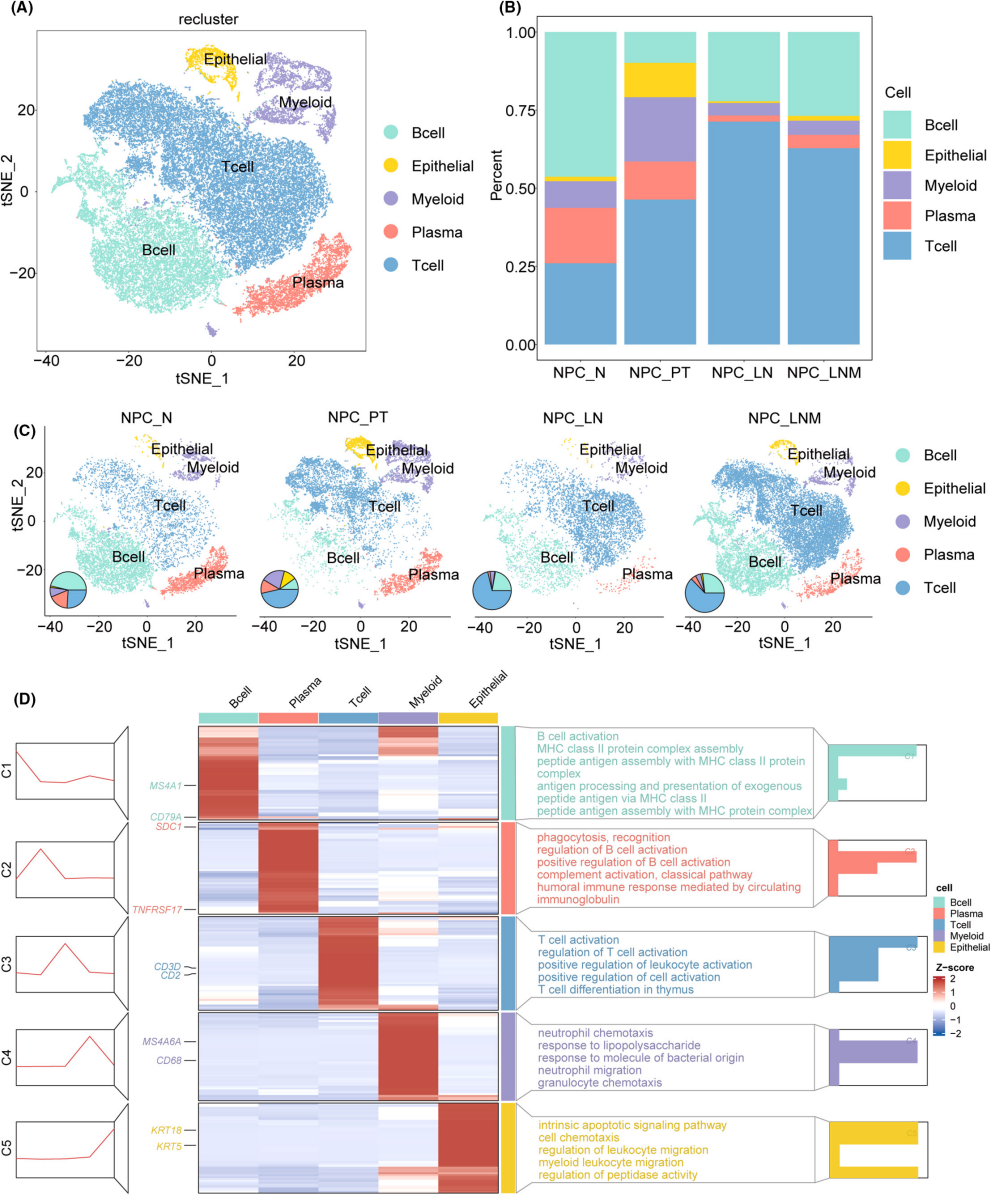
The single‐cell map of NPC in normal tissue, primary tumour, metastatic and normal lymph node ecosystem. (A) tSNE plot of 47,618 cells of NPC ecosystems, coloured by the major cell lineage. (B) The proportion of cell components of different NPC status. (C) tSNE plot and cell proportion of each major cell lineage across four distinct NPC ecosystems. (D) A heatmap shows the marker genes that are differentially expressed in major cell lineage, the functional enrichment results are shown in the right panel.

To construct a global NPC lymph node metastatic niche atlas, we collected single‐cell RNA sequencing from eight human projects, including NPC_PT (2 samples), NPC_N (1 sample), NPC_LNM (4 samples) and NPC_LN (1 sample). After batch‐effect processing and quality control, a total of 47,618 cells were obtained from these samples (Figure S1A). To characterize the NPC lymph metastatic ecosystem at the single‐cell level, we identified 13 transcriptionally distinct clusters with the top 20 principal components and visualized them with a t‐distributed stochastic neighbour embedding (tSNE) plot (Figure S1B). Based on the well‐known marker genes, five major cell types were annotated, including B cells, epithelial cells, myeloid cells, plasma cells and T cells (Figure 1A). Specific expression patterns of marker genes could be observed in corresponding cells. For example, B cells expressed CD79A and MS4A1, epithelial expressed KRT5 and KRT18, myeloid cells expressed CD68 and MS4A6A, plasma cells expressed TNFRSF17 and SDC1, T cells expressed CD2 and CD3D (Figure S1C).

We next compared the fraction of the five major cell types between four NPC statuses. The proportion of cell types was dynamic, which suggested the heterogeneity and complexity of tumour ecosystems in NPC metastasis (Figure 1B,C). B cells were predominated in normal nasopharynx tissue. Epithelial and myeloid cells were enriched in primary tumour samples. Compared between primary tumour and normal samples, the expansion of T cells, epithelial and myeloid cells and decreased of plasma cells were observed. Increased B cell and decreased T cell proportion were found in lymph nodes with metastasis compared to the normal lymph node sample. Functional enrichment analysis based on the DEGs further explains the distinct biological roles of major cell types. For example, the genes highly expressed in B cells enriched in B cell activation and antigen processing and presentation processes. T cell‐specific genes were significantly enriched in T cell activation and differentiation (Figure 1D). Taken together, we provided a comprehensive single‐cell map of the major cells in the NPC lymph node metastasis.

### 3.2 Diversity and function of T cells in NPC metastasis

**FIGURE 2:**
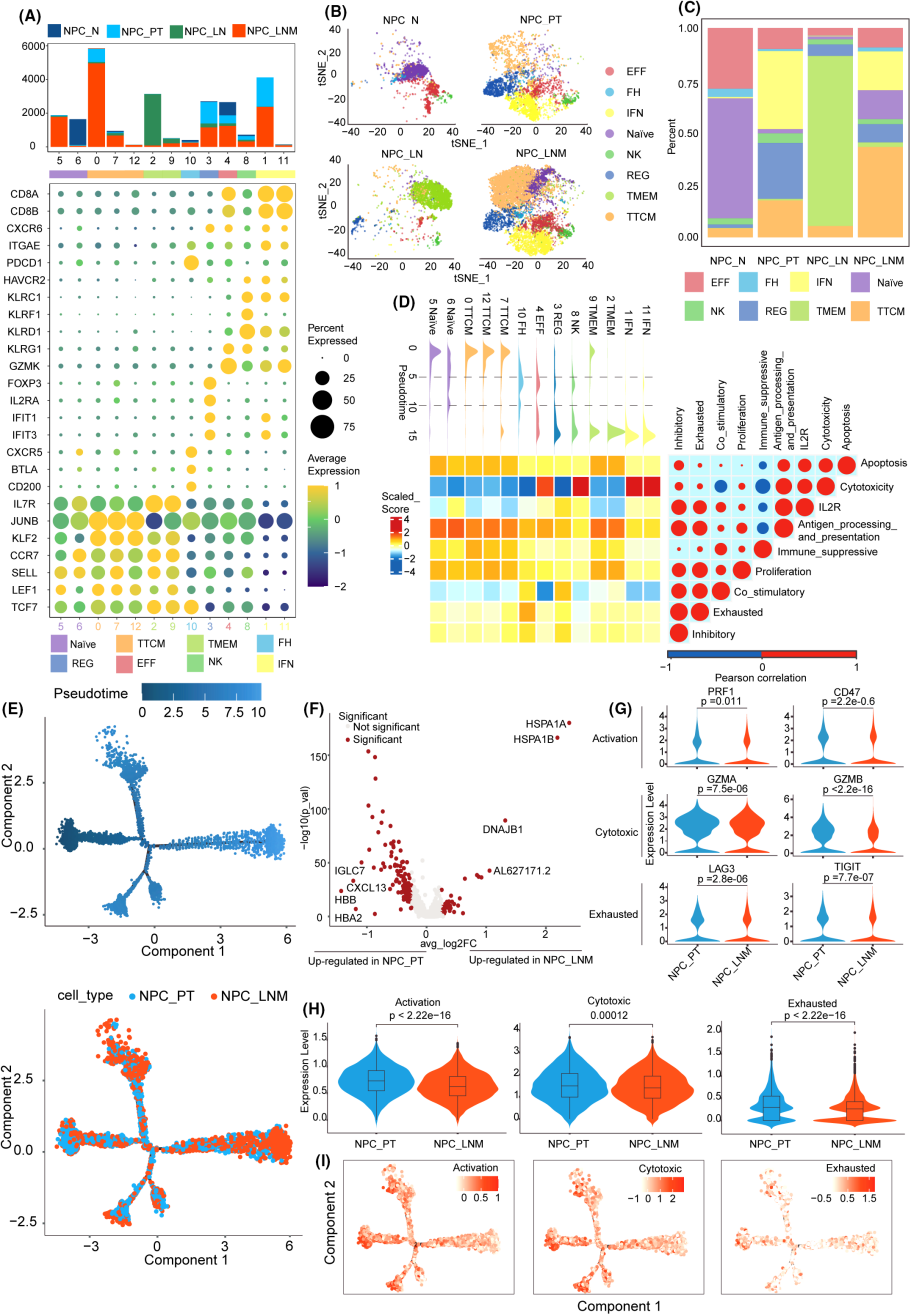
Distinct states of T cells of NPC metastatic ecosystem. (A) Bubble plot showing the differential expression of marker genes for T cell subclusters. The bar plot showing the cell number for each cluster, which is coloured by their originated ecosystem. (B) tSNE plot and cell proportion of each T cell lineage across four distinct NPC ecosystems. (C) The proportion of cell components of different NPC status. (D) Heatmap showing the dynamic changes in functional module scores along the pseudotime (upper panel) of T cell subtypes. (E) Pseudotime‐ordered analysis of CD8 T cells from PT and LNM samples. (F) Volcano plot showing the differentially expressed genes between LNM versus PT for CD8 T cells. (G) and (H) Comparison of the expression level and module scores of activation, cytotoxic and exhausted‐related genes between LNM and PT CD8 T cells with the Wilcoxon test. (I) Pseudotime plot showing the functional module scores along the trajectory.

A recent study has reported that the antitumor response of T cells is disrupted in metastatic lymph nodes, thus affecting the immunotherapy effect for human head and neck squamous cell carcinomas.^5^ To explore the potential roles of different T cell subtypes on NPC metastasis, we extracted 23,885T cells for further analysis. Using the cell subtype identification pipelines described in the Section 2, we subclustered the T cell population into 13 subgroups (Figures S2A and S2B). The canonical markers related to the phenotype of T cells were collected from Jia et al. work.^8^ Based on the average expression and expressed rate of cell markers, we clustered and annotated T cells into Naïve cells (C5 and C6), central memory T‐cell (TTCM, C0, C7 and C12), memory T cell (TMEM, C2 and C9), T follicular helper cells (FH, C10), regulatory T cell (REG, C3), effector T cells (EFF, C4), nature killer cells (NK, C8) and interferon‐induced T cells (IFN, C1 and C11) (Figure 2A). The information of cell markers and corresponding T cell subtypes is provided in Table S2. TTCM shared several common markers with naïve cells like SELL and TCF7 but expressed higher levels of JUNB and KLF2, which are consistent with the results of the previous study.^8^ The enrichment of the top 50 genes of each T cell subtype also supports our cell subclustering (Figure S2C).

Next, we compared the distribution of T cell subtypes in the different tumour ecosystems. Results showed that TMEM was predominated in NPC_LN, where naïve and EFF were enriched in NPC_N and decreased in NPC_PT and NPC_LNM (Figure 2B,C). Notably, IFN T cells exist in the NPC_PT and NPC_LNM samples, where their highly expressed IFIT1 and IFIT3 genes have been proven to be associated with tumour growth and metastases.^21^ Monocle2 analysis inferred a trajectory of T cells initiated at naïve state and TTCM, intermediated at FH, EFF, REG and finally terminated in TMEM and IFN status (Figure 2D). We found the functional module scores exhibited significant differences along with pseudotime (Kruskal–Wallis test, *p* < 0.05). Moreover, the positive correlation between cytotoxicity and APC, exhausted and inhibitory, IL2R and APC functional modules were observed in T cells (Figure 2D, *Rho* >0.7, FDR <0.05).

Since the initial activation of CD8 T cells in lymph nodes is essential for tumour immunotherapies,^22^ we next focused on the CD8 cells of NPC with lymph node metastasis. The CD8 cells of NPC_PT and NPC_LNM samples were first extracted from the Seurat object based on the well‐known markers CD8A, CD8B and GZMH (Figure S3A). The developmental trajectory of CD8 T cells was inferred via Monocle2 (Figure 2E). We found the T cell activation and defence response to the virus was enriched in the initial phase of the pseudotime (Figure S3B), which indicated the progressive loss of function of CD8 T cells during NPC metastasis. Consistent with these findings, the GSEA analysis based on the DEGs of CD8 T cells between NPC_LMN and NPC_PT revealed that the negative regulation of T cell‐mediated cytotoxicity is increased in NPC_LNM, while the response to virus and interferon‐gamma processes are decreased (Figure 2F and Figure S3C). Essential genes related to CD8 activation (PRF1 and CD47), cytotoxic (GZMA and GZMB) and exhausted (LAG3 and TIGIT) were all downregulated in NPC_LNM (Figure 2G). Moreover, the functional module scores exhibited the same trends as gene expression (Figure 2H,I). Collectively, these results indicated that NPC with lymph node metastasis evades CD8 T cell surveillance by immunosuppression.

### 3.3 Characteristics of myeloid cells in the NPC metastatic microenvironment

**FIGURE 3:**
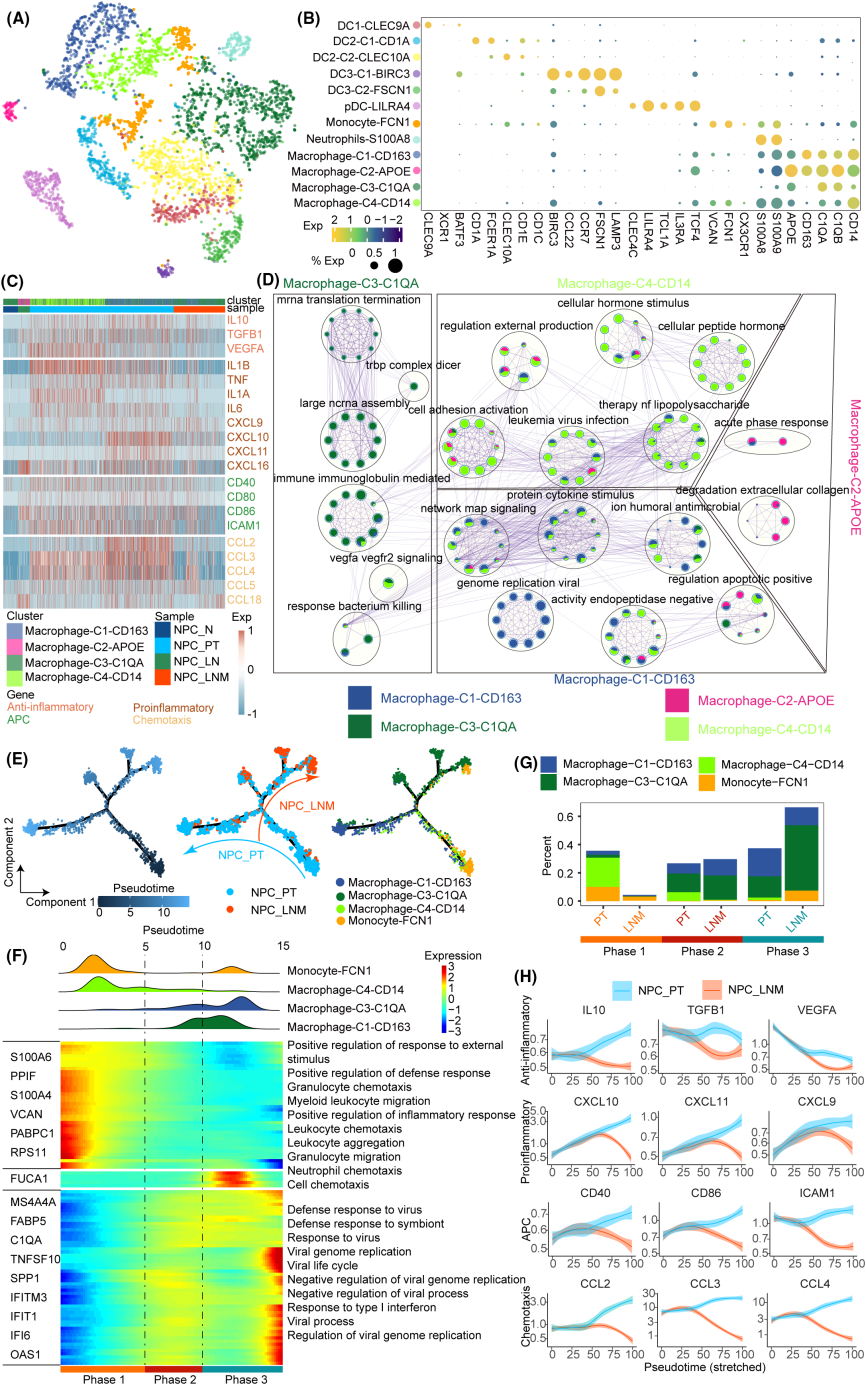
Characteristics of myeloid cells in the NPC metastatic microenvironment. (A) tSNE embedding plot of 4199 myeloid cells grouped into 12 clusters. (B) A dot plot showing marker gene expression across myeloid clusters from the adjacent tSNE plot. (C) Heatmap showing the expression level of anti‐inflammatory, proinflammatory, APC and chemotaxis‐related genes across macrophage subtypes and NPC status. (D) Functional enrichment results of macrophage subtypes. (E) Pseudotime‐ordered analysis of macrophage cells from PT and LNM samples. (F) Gene expression dynamics along the trajectory of macrophage cells. The distribution of macrophage subtypes during the transition (divided into three phases), along with the pseudo‐time. (G) Histogram showing the cell distribution of macrophage cells in LNM and PT samples. (H) Two‐dimensional plots showing the dynamic expression of essential genes in LNM and PT samples, along with the pseudotime.

The distinct tumour‐associated myeloid cells exhibited anti‐tumorigenic and pro‐tumorigenic phenotypes in cancer development and metastasis.^23^ To reveal the potential roles of myeloid cells in NPC lymph node metastasis, we identified 12 clusters (4199 cells) including six dendritic cells (DC) subsets (DC1‐CLEC9A, DC2‐C1‐CD1A, DC2‐C2‐CLEC10A, DC3‐C1‐BIRC3, DC3‐C2‐FSCN1 and pDC‐LILRA4), 1 monocyte cluster (Monocyte‐FCN1), 1 neutrophils cluster (Neutrophils‐S100A8), and 4 macrophage clusters (Macrophage‐C1‐CD163, Macrophage‐C2‐APOE, Macrophage‐C3‐C1QA and Macrophage‐C4‐CD14) (Figure 3A and Figure S4A). The marker genes of each cell type are shown in Figure 3B. Functional enrichment results further support the accuracy of the subcluster identification. For example, the top 50 expressed genes of DC2 are related to the MHC II protein complex, and genes of neutrophils are enriched in neutrophil migration (Figure S4B). The cellular composition results showed that DC2 and monocytes are predominant in NPC_N and NPC_LN, and the proportion of macrophage is increased in NPC_PT and NPC_LNM (Figure S4C).

The functional module scores related to DC including APC, apoptosis, costimulation, DC differentiation and immune suppressive were calculated and compared between DC subtypes. We found the distribution of these scores exhibited significant differences among the DC1, DC2, DC3 and pDC groups (Figure S5A). Notably, all these module scores are significantly decreased in the DC subtypes of NPC_LNM compared to NPC_PT, which indicated an immune‐suppressive microenvironment of NPC lymph node metastasis (Figure S5B). To further explore the dynamics of DC in NPC metastasis, we performed Monocle2 analysis and obtained the cell pseudo‐times along trajectories. We found the trajectory of DC initiated at pDC and differentiated into two branches (Figure S5C). The branch containing abundant NPC_PT cells is enriched DC2 cells, while the other branch composed of NPC_LNM is formed by DC1 and DC3 cells. Collectively, these data show the dynamic changes of DC subtypes in NPC metastasis.

For macrophage, we found the cells in NPC_N and NPC_LN are mainly C3‐C1QA and C2‐APOE, respectively. We found the genes and functional module scores including anti‐inflammatory, pro‐inflammatory, APC and chemotaxis are all increased in NPC primary tumour cells, while restrained in the lymph nodes with metastasis (Figure 3C and Figure S6A). The C1‐CD163 cluster was specifically enriched in the viral genome and shared several signalling processes with C4‐CD14. C2‐APOE was found to be involved in acute phase response and degradation of extracellular collagen. The function of C3‐C1QA was related to immune regulation and VEGFA signalling (Figure 3D). We further performed the developmental trajectory analysis based on the monocyte and macrophage cells for NPC_PT and NPC_LNM samples. Similar to DC, the trajectory differentiates into two different branches alongside NPC_PT and NPC_LNM (Figure 3E). We observed the trajectories initiated at monocyte and macrophage‐C4‐CD14 and finally terminated in the macrophage‐C1‐CD163 and macrophage‐C3‐C1QA for NPC_PT and NPC_LNM, respectively. We next investigated the transcriptional changes associated with trajectory and identified three phases (Figure 3F). Monocyte and macrophage‐C4 were predominantly phase 1 cells, characterized by the upregulated expression of NPC oncogenes like S100A6^24^ and PABPC1.^25^ Macrophage‐C1 and macrophage‐C3 were predominantly phase 3 cells, featured by several essential genes related to the tumour lymph nodes metastasis including FABP5,^26^ TNFSF10,^27^ SPP1^28^ and IFITM3.^29^ Pathway analysis indicated that the myeloid leukocyte migration and several chemotaxis signalling were enriched in phase 1, and pathways related to viral infective processes were enriched in phase 3 (Figure 3F). We found the proportion of lymph node metastatic cells gradually increased from phase 1 to phase 3, with a distinct distribution of macrophage subtypes (Figure 3G). The elevated expression of genes related to macrophage functional modules was observed in NPC_PT samples, whereas these genes exhibited decreased trends with the pseudotime for NPC_LNM (Figure 3H). These results further demonstrated the immunosuppressive myeloid microenvironment of lymph node metastasis, which is consistent with the findings of a previous study.^30^

### 3.4 Characterized B‐plasma cells in NPC metastasis

**FIGURE 4:**
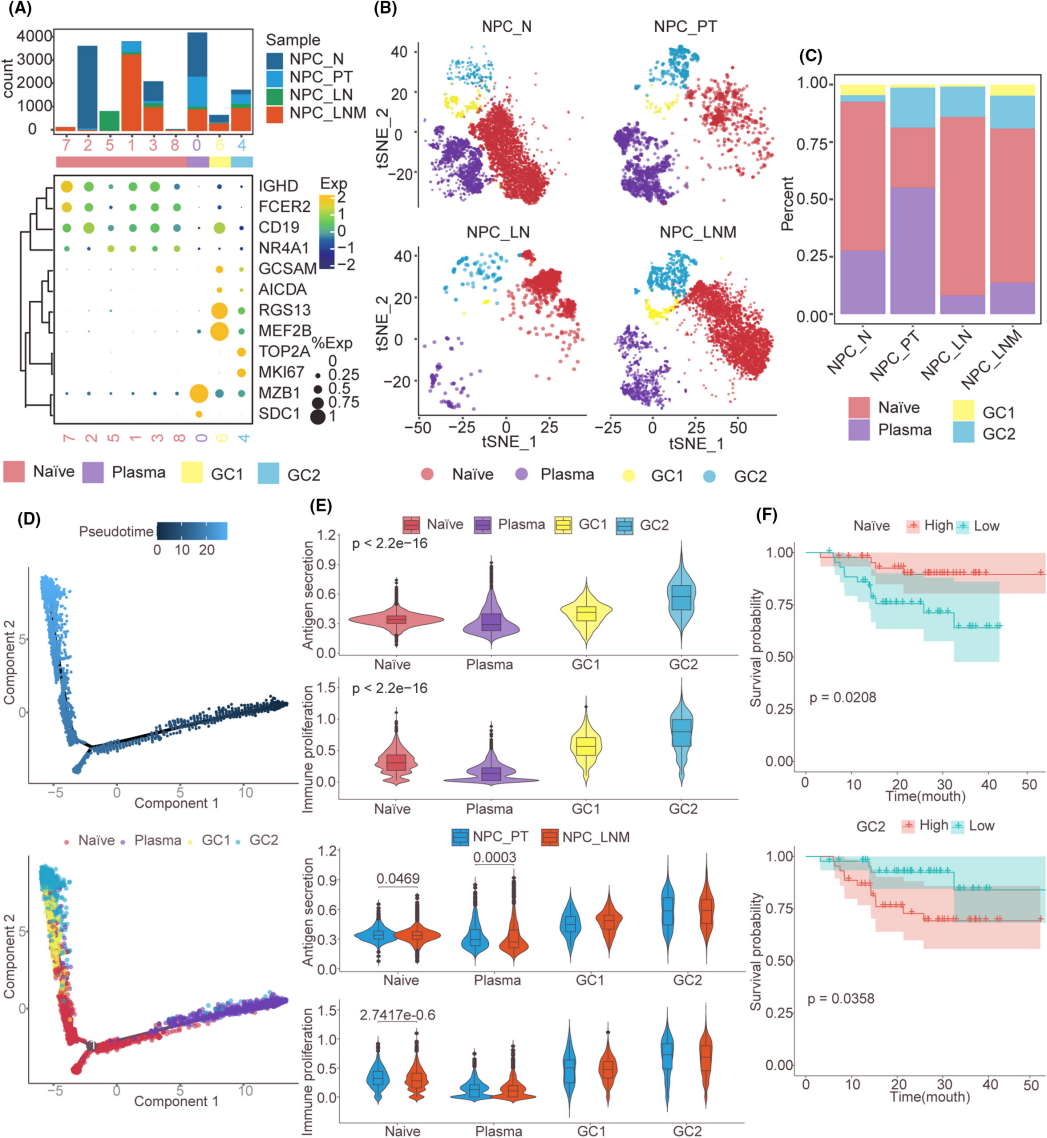
B cell clusters in NPC metastasis. (A) Bubble plot showing the differential expression of marker genes for B cell subclusters. The bar plot showing the cell number for each cluster, which is coloured by their originated ecosystem. (B) tSNE plot and cell proportion of each B cell lineage across four distinct NPC ecosystems. (C) The proportion of cell components of different NPC status. (D) Pseudotime‐ordered analysis of B cells from PT and LNM samples. (E) Comparison of antigen secretion and immune proliferation scores among B cell subtypes and NPC status. (F) Kaplan–Meier curves for progression‐free survival in the 113 NPC patients according to the high versus low scores of the cell marker signature.

B cells induced by primary breast tumour will selectively promote the lymph node metastasis,^31^ we next characterize the ecosystem of B‐plasma cells in NPC metastasis to explore their potential roles. Using tSNE dimension analysis, 16,893 cells were clustered into nine groups (Figure S7A and S7B). Based on the expression of marker genes, we annotated C1, C2, C3, C5, C7 and C8 which expressed IGHD, FCER2, CD19 and NR4A1 as naïve B cells (Figure 4A). Geminal center‐1 (GC1) and geminal center‐2 (GC2) B cells were characterized by GCSAM, AICDA, RGS13, MEF2B and TOP2A, MKI67, respectively. C0 clusters expressed MZB1 and SDC1 were considered plasma cells. Cellular composition analysis indicates that the proportion of naïve B cells was decreased, and plasma cell was increased in NPC_PT compared to other samples (Figure 4B,C). We found the trajectory was initiated with plasma cells, intermediated at naïve B cells, and finally terminated with GC1 and GC2 B cells (Figure 4D). The functional modules scores including antigen secretion and immunological proliferation were exhibited significantly different among B cell subtypes, where GC2 displayed larger scores (Figure 4E). Compared between NPC_LNM and NPC_PT, antigen secretion and immunological proliferation scores are decreased in naïve cells, whereas antigen secretion scores decreased in plasma cells (Figure 4E). Using bulk RNA sequencing data of NPC patients with PFS information, we found that higher naïve B cell scores were associated with better prognosis and GC2 B cell scores were related to poor survival (Figure 4F), suggesting the abundance of B cell subtypes could be used to predict the progress of NPC patients.

### 3.5 The heterogeneity and prognostic efficacy of epithelial cells

**FIGURE 5:**
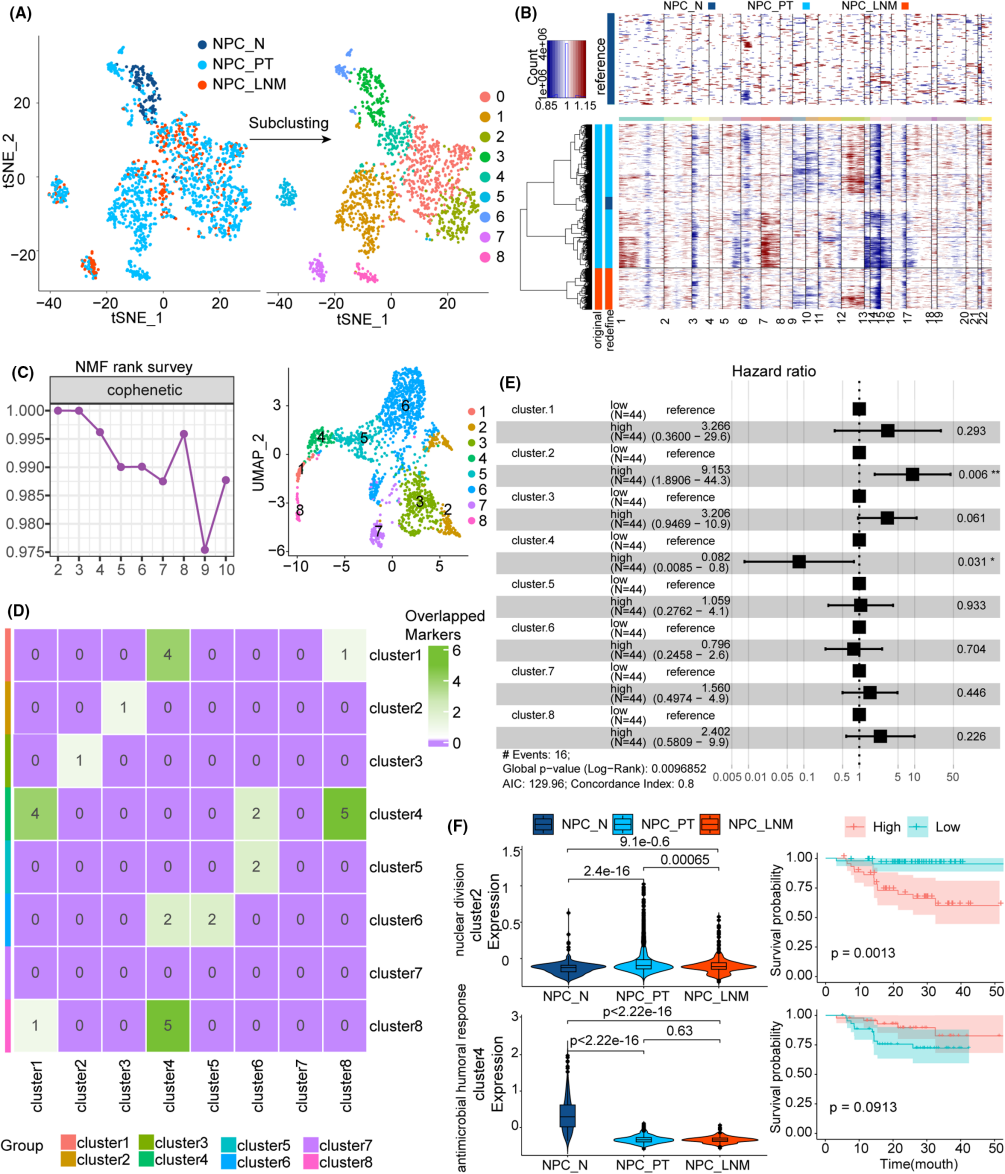
Heterogeneity of malignant epithelial cells in NPC. (A) tSNE plot of epithelial cells, coloured by originated ecosystem and subtypes. (B) Heatmap showing the large‐scale CNVs for epithelial cells (rows along the y‐axis) from normal nasopharynx tissue, primary tumour and metastatic lymph node ecosystems. Red: Amplification and blue: Deletion. (C) The NMF rank survey of inferred gene expression program numbers with the range of from 2 to 10. The tSNE plot of novel epithelial clusters inferred from NMF was shown in the right panel. (D) Heatmap showing the number of overlapped marker genes across epithelial cell subtypes inferred by NMF. (E) Prognostic values of epithelial clusters in the 113 NPC patients. Forest plots showing the Hazard rations and horizontal ranges derived from multivariate Cox regression survival analysis for progression‐free survival. (F) The violin plot showing the cell marker signature scores across the NPC ecosystem (left panel). Kaplan–Meier curves for progression‐free survival in the NPC cohort stratified according to the high versus low scores of the cell marker signature (right panel).

We next extracted 1610 epithelial cells (147 NPC_N, 1137 NPC_PT and 326 NPC_LNM) from the Seurat object to explore the heterogeneity of epithelial cells in NPC metastasis. The epithelial cells were clustered into nine groups, which indicated the existence of a diversity of epithelial cells (Figure 5A). Compared to the reference data of epithelial cells from normal nasopharynx tissue, the epithelial cells in primary and lymph node metastatic samples exhibited large‐scale copy number alteration, whereas 98 cells from NPC_PT were considered nonmalignant cells (Figure 5B). We observed a copy number deletions on chromosomes 3p and 14q, which is consistent with previous studies that the loss of tumour suppressor genes located in chromosomes 3 and 14 are frequently detected in NPC.^32^, ^33^

The top 50 DEGs between subclusters were identified and a large number of DEGs were shared between several subclusters, which suggested the homogeneity features hidden by gene expression profile (Figure S8). To fill these gaps, we performed an NMF algorithm on the expression matrix of epithelial cells as described in a previous study,^8^ and identified eight novel clusters with distinct gene expression programs based on the quality measures obtained from factorization rank (Figure 5C). The low number of overlapped DEGs between novel clusters demonstrated the high heterogeneity of epithelial cells in gene expression (Figure 5D). We found these clusters play different functional roles in the NPC metastasis (Figure S9A). For example, cluster1 was related to complement‐dependent cytotoxicity whose score decreased in NPC_PT and NPC_LNM, and cluster6 was involved in a cytokine‐mediated signalling pathway that accumulatively increased in NPC_PT and NPC_LNM (Figure S9B). A recent study reported that cancer cells could trigger sentinel lymph node remodelling by promoting the release of immunosuppressive cytokines of TIME,^34^ which is consistent with our findings. The multivariate Cox regression analysis of eight clusters based on NPC bulk cohort revealed the significant prognostic efficacy of cluster 2 and cluster 4 (Figure 5E, Figure S9C). The cluster 2 scores (most enriched in nuclear division) were upregulated in NPC_PT and NPC_LNM, which could serve as a risky factor in NPC survival (Figure 5F). Conversely, the cluster 4 scores (most enriched in antimicrobial humoral response) were decreased in NPC_PT and NPC_LNM, whose higher scores could predict better survival for NPC patients (Figure 5F).

### 3.6 Intercellular communications of NPC primary and metastatic ecosystems

**FIGURE 6:**
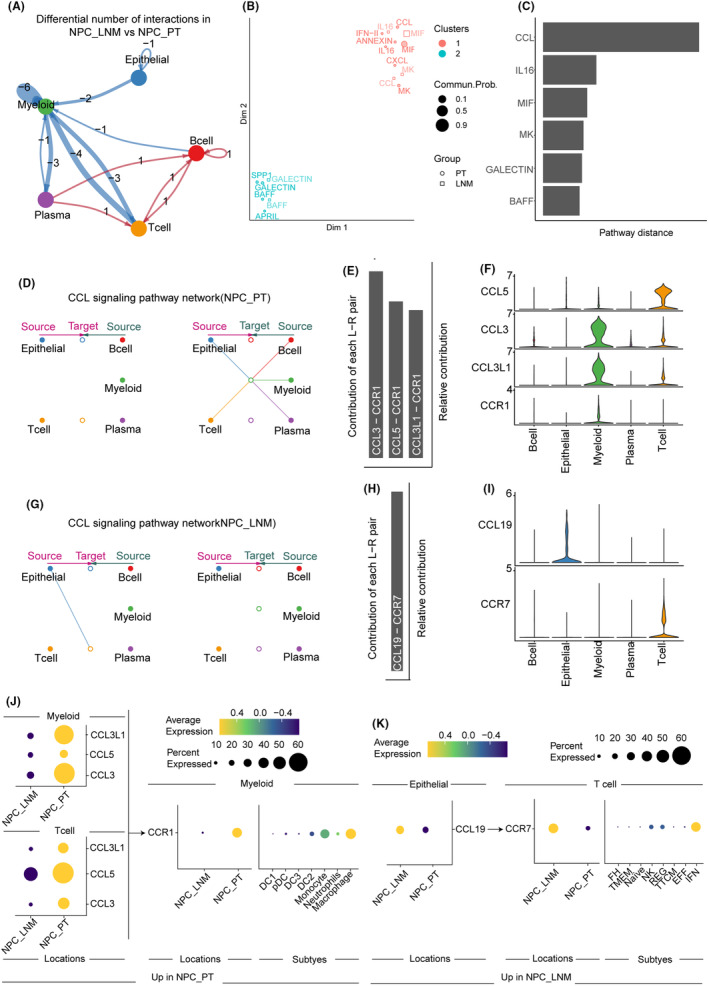
Comparison analysis of cell–cell communications between LNM and PT of NPC patients. (A) Circle plot shows the difference of cell communications for LNM versus PT in NPC. Red edges represent increased signalling and blue edges represent decreased signalling. (B) Jointly projecting and clustering signalling pathways from LNM and PT of NPC patients into a shared 2‐dimensional manifold according to their functional similarity. (C) The overlapping pathways were ranked based on their pairwise Euclidean distance in the shared 2‐dimensional manifold. (D) The inferred CCL signalling network in primary tumour. The left and right portions show autocrine and paracrine signalling, respectively. (E) The relative contribution of each ligand–receptor pair to the overall CCL signalling network in primary tumour. (F) Expression level of CCL signalling genes in the primary tumour. (G) The inferred CCL signalling network in lymph node metastasis. (H) The relative contribution of each ligand–receptor pair to the overall CCL signalling network in lymph node metastasis. (I) Expression level of CCL signalling genes in lymph node metastasis. (J) and (K) Dot plot of the differential expression of signalling ligand–receptor pairs by compartment and cell types in primary tumour and lymph node metastasis ecosystem.

To explore the mechanism of cell–cell interactions in NPC lymph node metastasis, we inferred the intercellular communications for NPC_PT and NPC_LNM separately based on CellChat (Figure S10A). We observed the myeloid‐related interactions with other cells were reduced in lymph node metastasis compared to primary tumours, while communications among plasma, B cells and T cells were increased (Figure 6A). In total, there were eighteen L‐R pairs decreased in NPC patients with lymph node metastasis, and the communication probability of five pairs including MIF‐(CD74 + CXCR4), MIF‐(CD74 + CD44), LGALS9‐CD45, IL16‐CD4 and CCL19‐CCR7 were increased in NPC_LNM (Figure S10B,C). The signalling pathways associated with inferred communications were next mapped onto a 2‐dimensional manifold and clustered into two groups (Figure 6B). Several cytokine‐related including IL16 and IFN‐II were grouped into cluster1, while cluster2 were involved in immune and inflammatory‐related signalling like GALECTIN and BAFF.

The Euclidean distance of the shared signalling pathways between NPC_PT and NPC_LNM was calculated (Figure 6C). The maximum distance of the CCL pathway was observed, and we next specifically examined how CCL communications change from NPC_PT to NPC_LNM (Figure 6D–I). In primary tumours, myeloid cells were the only receptors of CCL, receiving the signals from all cells including epithelial, myeloid, plasma, B and T cells. However, the communications were sharply reduced in lymph nodes with metastasis, only interactions between epithelial cells and T cells were observed. The CCL3/CCL5/CCL3L1‐CCR1 axis contributed to the communications of CCL signalling in NPC_PT, which exhibited higher expression in myeloid and T cells. Instead, the CCL19‐CCR7 L‐R pair was the only interaction of CCL signalling in NPC_LNM, which is expressed in epithelial and T cells. Consistent with these results, we found the expression of the ligands (CCL3, CCL5 and CCL3L1) were significantly upregulated in myeloid and T cells for NPC_PT samples, and the expression of receptor CCR1 was increased in the macrophage subtypes of myeloid cells (Figure 6J). In NPC_LNM, the ligand CCL19 and receptor CCR7 were all upregulated in the epithelial and T cells for NPC_LNM samples, respectively. The IFN T cell subtypes exhibited a higher expression of CCR7 (Figure 6K). A recent study reported that the tumour‐derived CCL19 was associated with lymph node metastasis and suppresses the CD8 T cell function, which was similar with our results.^35^ Collectively, the multifaceted assessment of intercellular communications enables the finding of dynamic patterns from NPC_PT to NPC_LNM.

## 4 DISCUSSION

Single cell‐sequencing technology provided opportunities to deeply understand the tumour ecosystems, which facilitated the development of individualized treatment of tumours. Although the TIME of primary NPC has been systematically described in previous studies at single‐cell resolution,^11^, ^36^ the tumour ecosystems of lymph node metastasis in NPC patients remain unclear. In this study, our integrated single‐cell transcriptome analysis yields a comprehensive map of NPC lymph node metastasis that captured the major subsets of tumour and immune compartments. Through comparing analysis between primary and lymph node samples, we observed an immunosuppression microenvironment of metastatic lymph nodes for NPC patients, which is consistent with previous study that the immunosuppressive lymph node assists tumour colonization and growth.^4^

In particular, the negative regulative T cell‐mediated cytotoxicity and other immunosuppression‐related functions were enriched in the CD8 T cells at lymph node metastasis. Pseudo‐time analysis of T cells reveals that the end in an IFN T cell status with the high expression level of IFIT1 and IFIT3, consistent with previous understanding that IFN signalling in cytotoxic T cells restrict anti‐tumour immunity by inhibiting the maintenance, clonal diversity and proliferation of stem‐like T cells in metastatic melanoma patients.^37^ In addition, IFIT1 and IFIT3 have been reported to promote the lymph node metastasis of HNSC.^21^ Zhang et al. identified that C1QA/B+ macrophages exert central regulatory roles for immune evasion of oesophageal squamous cell carcinoma with liver metastasis.^38^ In our results of myeloid cells, we also recognized the macrophage C3 that is defined by a higher expression of C1QA and C1QB. The proportion of macrophage C3 was aggressively increased with the developmental trajectory, especially enriched in lymph node metastatic cells (from phase 1 to phase 3, Figure 3G). These results are consistent with the knowledge that the synthesizing of complement C1q in the TIME could act as a protein of the extracellular matrix to promote tumour metastasis.^39^ B cells play complex roles in tumour immunity that are involved in both promoting and inhibiting tumour metastasis.^40^ Previous study has demonstrated that the naïve B cells were more enriched in tumour‐draining lymph nodes which exhibited immunosuppressive phenotypes for HNSC.^41^ Our results also observed an increased percentage of naïve B cells in NPC_LNM, while the antigen secretion and immunological proliferation scores were decreased compared to NPC_PT cells. Collectively, these results revealed the immunosuppressive immune cell microenvironment in the metastatic lymph nodes which may promote the tumour metastasis of NPC patients.

Cell–cell communication analysis also reveals the dynamic signalling among different cell types in NPC metastasis. We identified CCL pathways that showed the maximum pathway distance between NPC primary and lymph node metastatic cells. We found that CCL3 (also known as MIP‐1α) and its receptor CCR1 were highly expressed in myeloid cells for NPC primary patients. It has been demonstrated that CCL3 was detected in the CD68^+^ cells that infiltrated in NPC biopsies while exhibiting low levels in controls, which is consistent with our results that CCL3 was activated in the TIME of NPC primary tissues.^42^ We also observed the upregulated CCL5 in NPC primary cells. Epstein–Barr virus (EBV) infection, a factor consistently associated with NPC carcinogenesis, could increase the expression of CCL5 to induce tumour angiogenesis and growth.^43^ We identified a different CCL L‐R pair (CCL19‐CCR7) that may be involve in NPC lymph node metastasis. The CCL19 and its receptor CCR7 were specifically expressed in epithelial cells and IFN T cells of NPC_LNM, respectively. Consistent with the previous understanding that tumour‐derived CCL19 inhibited the CD8 T cell and promoted lymph node metastasis,^35^ we hypothesize NPC tumour cells will release CCL19 and reshape the immunosuppressive TIME of metastatic lymph nodes via CCR7. These results highlight the essential roles of CCL signalling in both NPC occurrence and metastasis, which rely on the different L‐R pairs to rewire cell communications.

In conclusion, our study reveals the dynamic tumour ecosystems that are closely associated with NPC lymph node metastasis. We observed global immunosuppressive microenvironments of lymph nodes which may support the tumour cell growth and metastasis. Further cell–cell communications highlight the essential pathways in NPC metastasis. These results can aid in the development of personality therapy for NPC patients with lymph node metastasis.

## AUTHOR CONTRIBUTIONS

**Dahua Xu:** Data curation (equal); formal analysis (equal); investigation (equal); methodology (equal); validation (equal); visualization (equal); writing – original draft (equal); writing – review and editing (equal). **Nihui Zhang:** Data curation (equal); formal analysis (equal); investigation (equal); methodology (equal); validation (equal); visualization (equal). **Yutong Shen:** Data curation (equal); formal analysis (equal); investigation (equal); methodology (equal); validation (equal); visualization (equal). **Dehua Zheng:** Data curation (equal); formal analysis (equal); investigation (equal); methodology (equal); validation (equal); visualization (equal). **Zhizhou Xu:** Data curation (equal); formal analysis (equal). **Peihu Li:** Data curation (equal); formal analysis (equal). **Jiale Cai:** Data curation (equal); formal analysis (equal). **Guanghui Tian:** Data curation (equal); formal analysis (equal). **Qingchen Wei:** Data curation (equal); formal analysis (equal). **Hong Wang:** Data curation (equal); formal analysis (equal). **Hongyan Jiang:** Data curation (equal); formal analysis (equal). **Meng Cao:** Data curation (equal); formal analysis (equal); funding acquisition (equal); project administration (equal); supervision (equal). **Bo Wang:** Funding acquisition (equal); investigation (equal); methodology (equal); project administration (equal); supervision (equal). **Kongning Li:** Conceptualization (equal); funding acquisition (equal); project administration (equal); supervision (equal); writing – original draft (equal); writing – review and editing (equal).

## FUNDING INFORMATION

This work was supported by the National Natural Science Foundation of China (32160152), Major Science and Technology Program of Hainan Province (ZDKJ2021040), Hainan Provincial Natural Science Foundation of China (824QN271), Innovative research project for Graduate students in Hainan Province (Qhys2023‐489, Qhys2023‐447, HYYB2023A36, HYYB2023A06).

## CONFLICT OF INTEREST STATEMENT

The authors confirm that there are no conflicts of interest.

## Supporting information


Figures S1–S10.

Tables S1–S3.

## Data Availability

The public transcriptome profiles and clinical data were provided in Section 2. Software and resources used for the analyses are described in each Section 2.
