# Supplementary material for: Single‐cell sequencing analysis reveals the dynamic tumour ecosystems of primary and metastatic lymph nodes in nasopharyngeal carcinoma
Source: J Cell Mol Med. 2024 Oct 11;28(19):e70137. doi: 10.1111/jcmm.70137 (PMC11467730; doi:10.1111/jcmm.70137)
Supplement: Supplementary file 1 — Figures S1–S10. [file JCMM-28-e70137-s001.pdf]

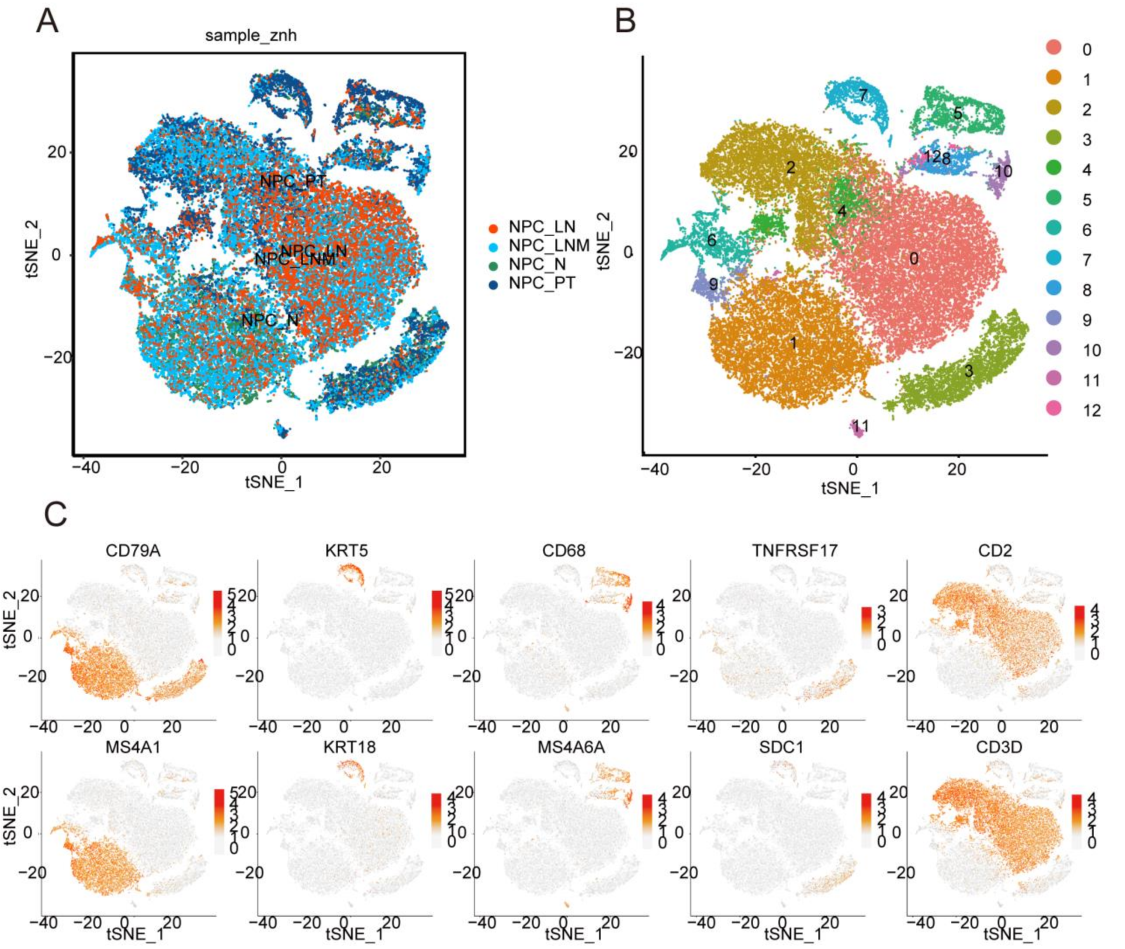

Supplementary figure1. Cell clusters of single cells of NPC ecosystem. (A) and (B) tSNE plot of 47,618 cells of NPC ecosystems, colored by originated ecosystem and subtypes. (C) t-SNE projection of single cells, with cells colored based on the expression of marker genes for major cell types.

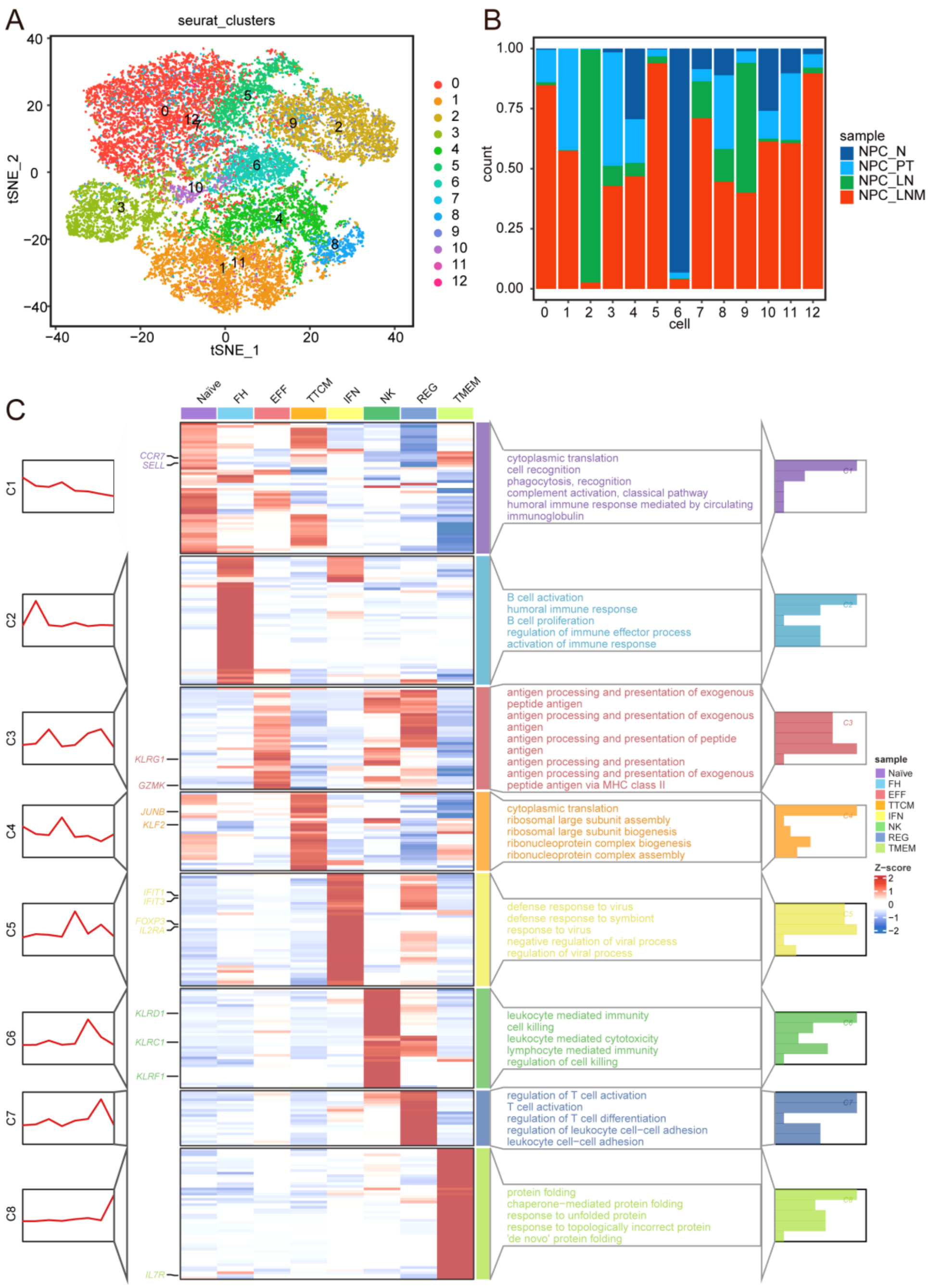

Supplementary figure2. Cell clusters of T cells of NPC ecosystem. (A) tSNE plot for T cells, colored by subtypes. (B) The proportion of T cell components of different NPC status. (C) A heatmap shows the marker genes that are differentially expressed in the T cell lineage, the functional enrichment results are shown in the right panel.

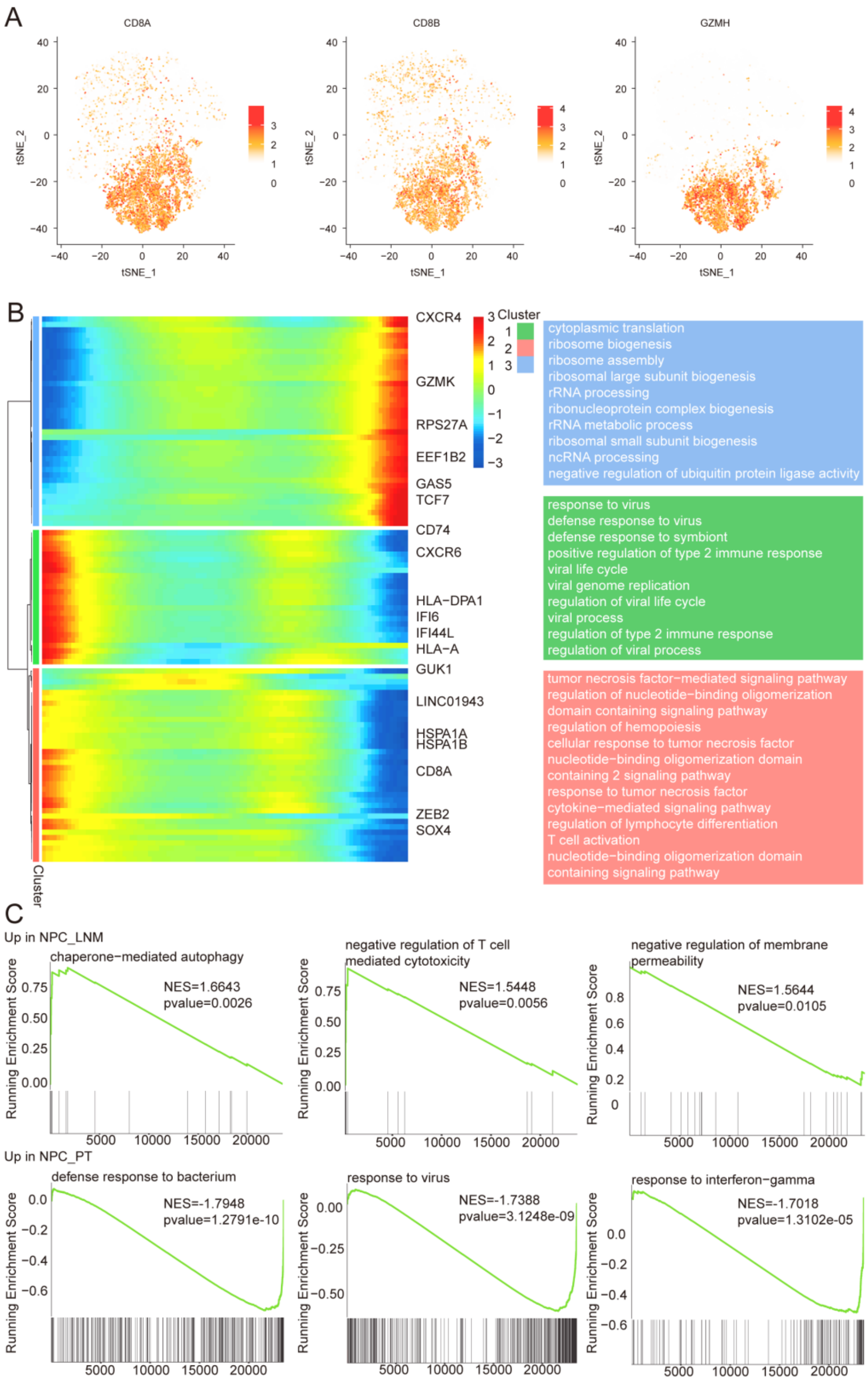

Supplementary figure3. Characteristics of CD8 T cells in the NPC ecosystem. (A) t-SNE projection of single cells, with cells colored based on the expression of marker genes for CD8 T cells. (B) Gene expression dynamics along the trajectory of CD8 T cells. (C) GSEA results for the differentially expressed genes for LNM vs PT.

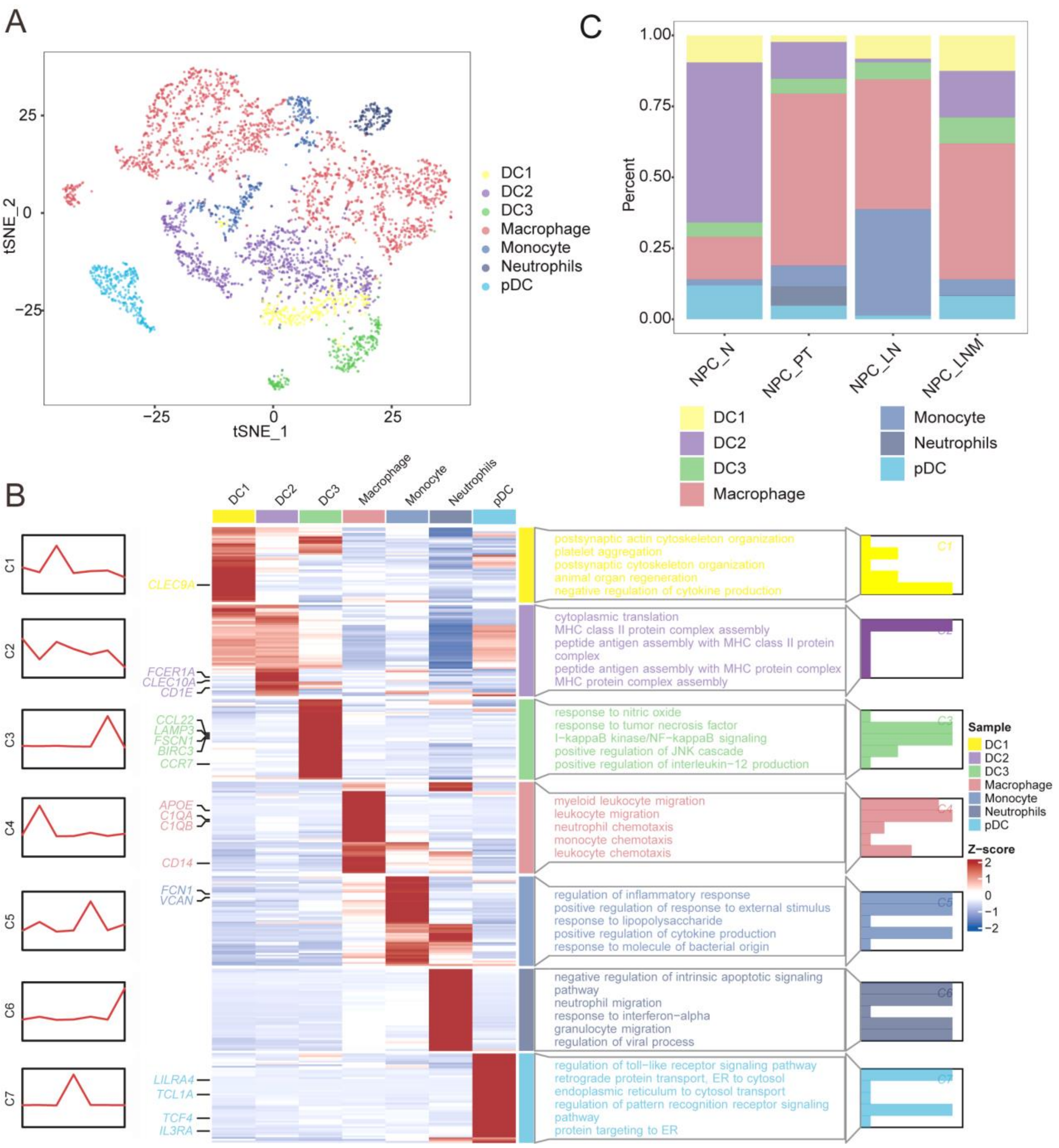

Supplementary figure4. Characteristics of myeloid cells in the NPC ecosystem. (A) t-SNE projection of myeloid cells. (B) The proportion of myeloid cell components of different NPC status. (C) A heatmap shows the marker genes that are differentially expressed in myeloid cell lineage, the functional enrichment results are shown in the right panel.

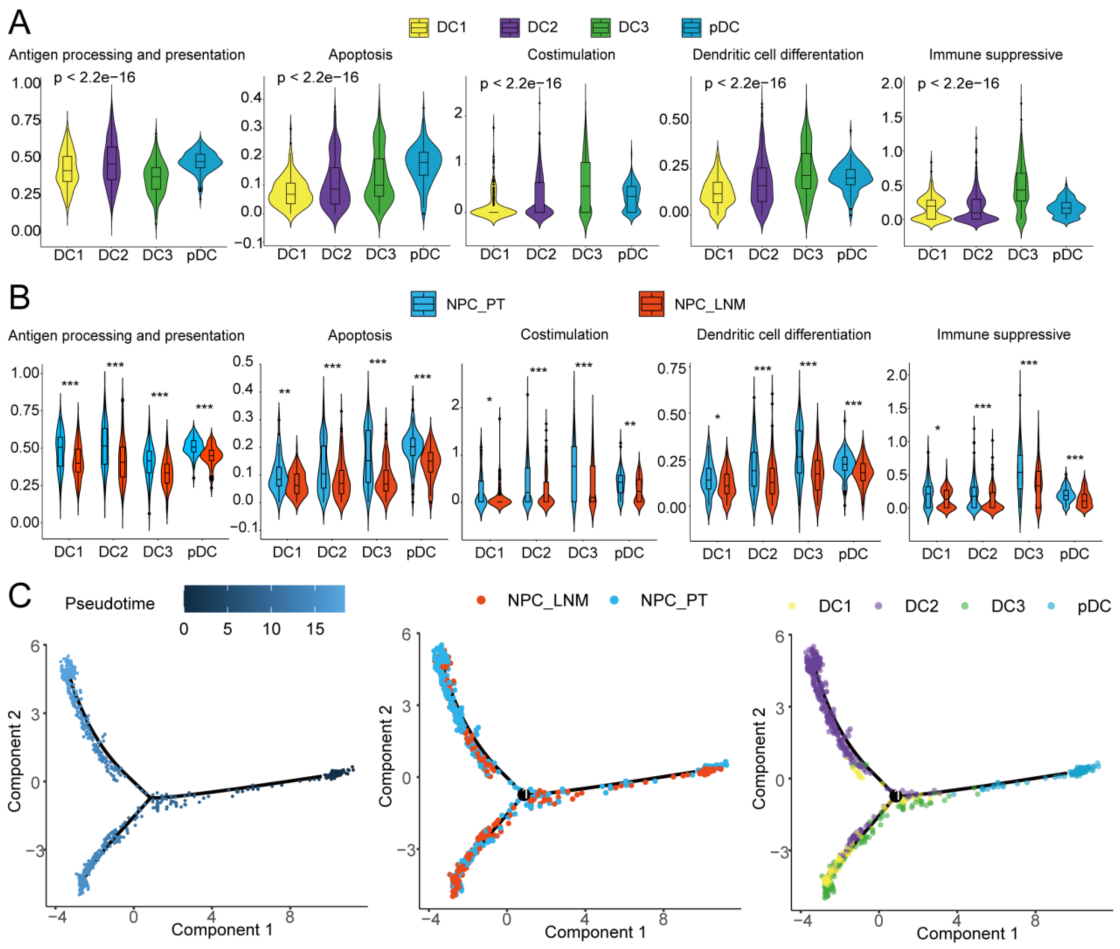

Supplementary figure5. Characteristics of dendritic cells in the NPC ecosystem. (A) Comparison of functional module scores among dendritic cell subtypes. (B) Comparison of functional module scores between LNM and PT of NPC patients. (C) Pseudotime-ordered analysis of dendritic cells from PT and LNM samples.

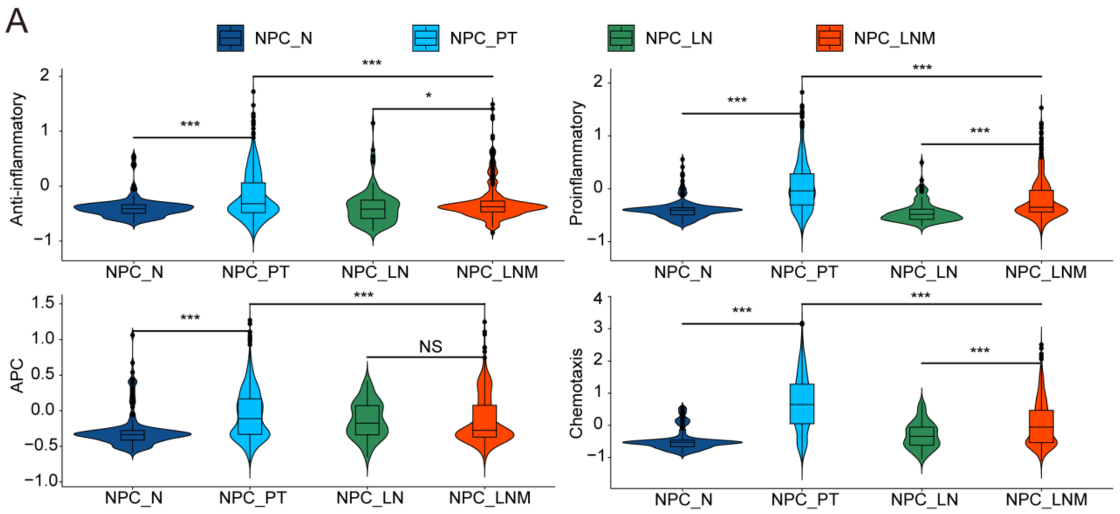

Supplementary figure6. Comparison of functional module scores of macrophage cells among NPC ecosystems.

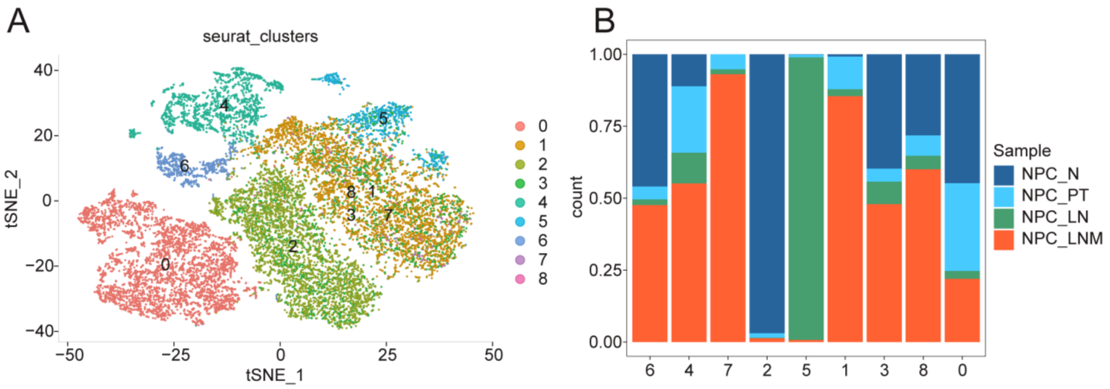

Supplementary figure7. Characteristics of B cells in the NPC ecosystem.

(A) t-SNE projection of B cells, colored by subtypes. (B) The proportion of myeloid cell components of different NPC status.

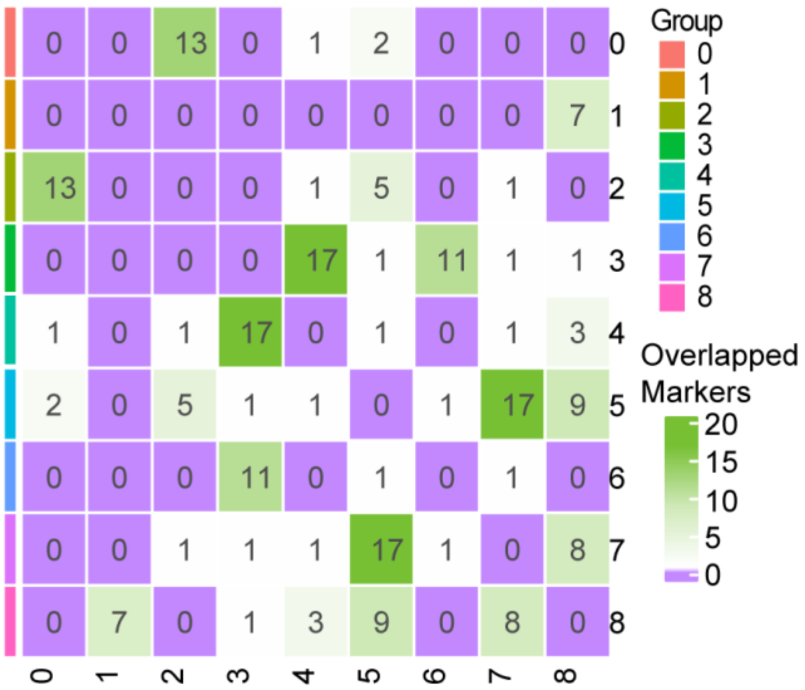

Supplementary figure8. Heatmap showing the number of overlapped marker genes across epithelial cell subtypes inferred by Seurat.

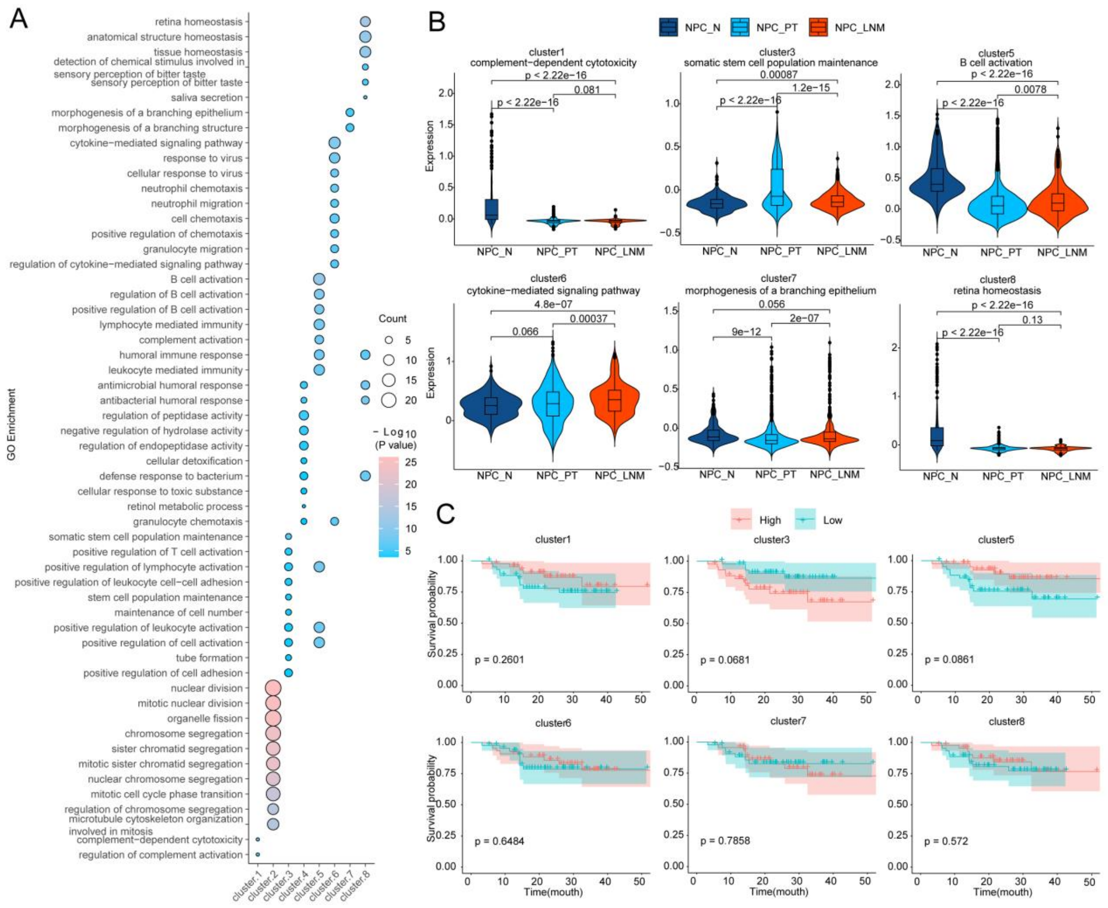

Supplementary figure9. Characteristics of epithelial cell clusters in NPC ecosystem. (A) The functional enrichment results of epithelial cell clusters were inferred by NMF. (B) The violin plot showing the cell marker signature scores across the NPC ecosystem. (C) Kaplan–Meier curves for progression-free survival in the NPC cohort stratified according to the high vs low scores of the cell marker signature.

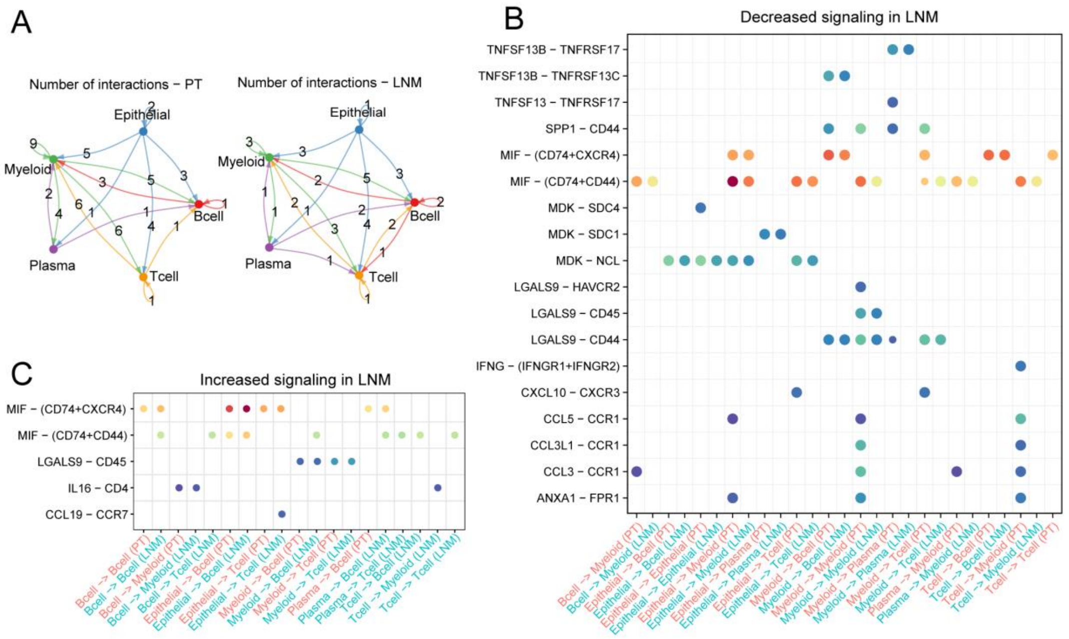

Supplementary figure10. Dynamic cell-cell communications between LNM and PT for NPC patients. (A) Circle plot shows the number of cell communications for LNM and PT in NPC. (B) and (C) The upregulated and downregulated signaling ligand–receptor pairs between lymph node metastasis and primary tumor cells.
